# Single-cell RNA sequencing of leukocytes at the maternal-fetal interface in physiological and pathological Nodal-deficient pregnancies

**DOI:** 10.3389/fimmu.2026.1611813

**Published:** 2026-04-16

**Authors:** Sarah Yull, Alain Pacis, Laura Girardet, Daniel Dufort

**Affiliations:** 1 Division of Experimental Medicine, McGill University, Montréal, QC, Canada; 2 Child Health and Human Development Program, Research Institute of the McGill University Health Centre, Montréal, QC, Canada; 3 Canadian Centre for Computational Genomics, McGill University, Montréal, QC, Canada; 4 Victor Phillip Dahdaleh Institute of Genomic Medicine, McGill University, Montréal, QC, Canada; 5 Department of Obstetrics and Gynecology, McGill University, Montréal, QC, Canada

**Keywords:** pregnancy, reproductive failure, fetal loss, intrauterine growth restriction, Nodal, macrophage, neutrophil, single cell RNA sequencing

## Abstract

Leukocytes at the maternal-fetal interface have critical functions during pregnancy in the prevention of maternal reactivity towards fetal alloantigens, suppression of excess inflammation and promotion of placental angiogenesis. Failed maternal immune adaptations to pregnancy can lead to placental insufficiency and the development of severe reproductive complications. Recent evidence from mouse models has suggested that Nodal, a secreted morphogen of the TGFβ superfamily, is an immunoregulator of pregnancy. The absence of Nodal expression in the female reproductive tract (Nodal^Δ/Δ^) resulted in the loss of preimplantation regulatory T cells and a 50% implantation failure rate. By mid-gestation, Nodal-deficient pregnancies showed placental dysfunction, fetal loss and intrauterine growth restriction. Therefore, the Nodal^Δ/Δ^ model represents a unique system to study immune-mediated reproductive failure. In this study, single-cell RNA-sequencing was used to characterize leukocytes within the mid-gestational decidua and placenta during physiological and pathological Nodal^Δ/Δ^ pregnancies. Eleven distinct immune cell clusters were identified, with an emphasis on myeloid populations (macrophages and neutrophils). In particular, the transcriptional characterization of placenta-associated maternal macrophages revealed functions consistent with the regulation of angiogenesis, immune suppression and endocytosis. In Nodal^Δ/Δ^ females, the differential abundance and expression profile of these leukocytes reflected the immune dysfunction observed in human reproductive pathologies. Taken together, elucidating roles of leukocytes in placental development provides a basis in the understanding of mechanisms associated with physiological pregnancy and reproductive failures.

## Introduction

Maternal immune adaptations during pregnancy are a critical component of reproductive success as the progression through implantation, placentation and parturition is partially regulated by tissue-resident leukocytes at the maternal-fetal interface. These changes are initiated during the preimplantation period by uterine regulatory T cells (Tregs), which restrict inflammation and establish maternal immunotolerance towards the semi-allogeneic blastocyst (1, 2). After implantation, the formation of the interface between the maternal decidua and fetal placenta coincides with the highest abundance of leukocytes during pregnancy. At this stage of placental angiogenesis, immune cells represent 30-40% of total cells and are mainly comprised of uterine natural killer cells (uNKs, 70%), macrophages (20-25%) and T cell subtypes (3-10%) (3, 4). In physiological pregnancy, uNKs and macrophages are associated with the production of cytokines, growth factors and matrix metalloproteinases (MMPs) that help mediate vascular endothelial cell breakdown, extravillous trophoblast invasion and spiral artery remodeling (5–8). Concurrently, immune suppression and evasion is sustained by Tregs and TGFβ signaling, which promotes alternative “M2” macrophage polarization and prevents cytotoxic uNK or T cell reactions to the fetus by inducing the activation of immune checkpoint molecules (1, 9, 10). Indeed, any deviation in leukocyte abundance or an expression profile that shifts from anti-inflammatory tolerance towards pro-inflammatory activation could lead to the development of severe complications such as fetal rejection, recurrent pregnancy loss (RPL, also known as recurrent spontaneous abortion or miscarriage), placental insufficiency, preeclampsia (PE), intrauterine growth restriction (IUGR) or preterm birth (PTB) (1, 5–8).

Nodal, a secreted morphogen of the TGFβ superfamily, was recently implicated as an immunomodulator of pregnancy (11, 12). Nodal expression increases from the uterine glandular epithelium of the mouse throughout the preimplantation period until implantation on day 4.5 (d4.5), when it becomes localized to regions between the implantation sites. After implantation, Nodal is minimally expressed in stromal cells of the anti-mesometrial decidua (13). The generation of a reproductive tract-specific Nodal knockout (Nodal^Δ/Δ^) mouse model (14) demonstrated the importance of Nodal expression in the establishment of pregnancy as 50% of Nodal^Δ/Δ^ females experienced implantation failure. Intriguingly, FOXP3^+^ Tregs were completely absent in all d3.5 Nodal^Δ/Δ^ uteri while inflammatory myeloid cells (macrophages, neutrophils) were more abundant. This suggested a role for preimplantation Nodal expression in the support of maternal immunotolerance similar to that of TGFβ (11).

For the 50% of Nodal^Δ/Δ^ females with implantation success, the emergence of mid-gestational reproductive failures revealed potential derivative effects of an altered preimplantation immune environment (11, 12, 14, 15). During placental development, on d10.5 Nodal^Δ/Δ^ females showed decreased decidual vascularization and impaired trophoblast differentiation. These deficiencies corresponded with significant fetal loss or IUGR for the few surviving embryos by d12.5 and PE-like phenotypes (14, 15). An increased risk for inflammation-induced preterm labour was also reported (12). In related human studies, an association between inflammation and maternal genetic polymorphisms that reduce Nodal bioactivity was described in both IUGR-complicated PE and as a risk factor for PTB (12, 16). Critically, recent hypotheses from human studies have also implicated the inadequate number or functional competence of Tregs as not only the basis of implantation failure but an underlying cause of reproductive failures that present clinically later in gestation (ie. RPL, PE, IUGR, PTB) (1, 17). Therefore, due to the similar, immunoregulatory function of Nodal at the maternal-fetal interface and the reproductive phenotypes observed in Nodal^Δ/Δ^ females, it is a suitable model to investigate the early pathogenesis of immune-mediated reproductive failure.

Single-cell RNA-sequencing (scRNA-seq) is a breakthrough technology used to study gene expression at the level of individual cells and demonstrate heterogeneity within complex populations. It has been applied to human peripheral blood, tissues from first trimester elective terminations and the placenta in contexts of physiological pregnancy, RPL and PE (18–30). However, the relevance of peripheral blood can be debated as it does not entirely reflect the local gestational environment (8, 31). Similarly, while most studies have emphasized trophoblast subtypes in the placenta, the contribution of maternal cells is often disregarded. Earlier mechanisms of reproductive failure are also not evident when analyzing the delivered placenta. Despite the benefit of mouse models to address these limitations in human reproduction, most murine scRNA-seq studies have also excluded cells of the maternal decidua to report just on the placenta under physiological conditions (32–34). Only a few late-gestational models of obesity, environmental stress and PTB have considered the role of maternal leukocytes in reproductive pathologies (35–37). Therefore, much remains to be discovered about maternal immune regulation in physiological pregnancy and at the onset of pathological complications.

To address these gaps in the understanding of physiological and pathological pregnancies, scRNA-seq was conducted on CD45^+^ cells at the d10.5 maternal-fetal interface of Nodal^loxP/loxP^ control and Nodal^Δ/Δ^ females prior to the emergence of fetal loss and IUGR. An emphasis was placed on the characterization of myeloid cells, including the novel identification of placenta-associated maternal macrophages (PAMMs) in the mouse at mid-gestation. PAMM expression profiles indicated a role in the regulation of angiogenesis and immune suppression during pregnancy, with PAMM and neutrophil dysregulation observed in the Nodal^Δ/Δ^ model of reproductive failure.

## Results

### Identification of eleven immune cell clusters at the d10.5 maternal-fetal interface by scRNA-seq

**Figure 1:**
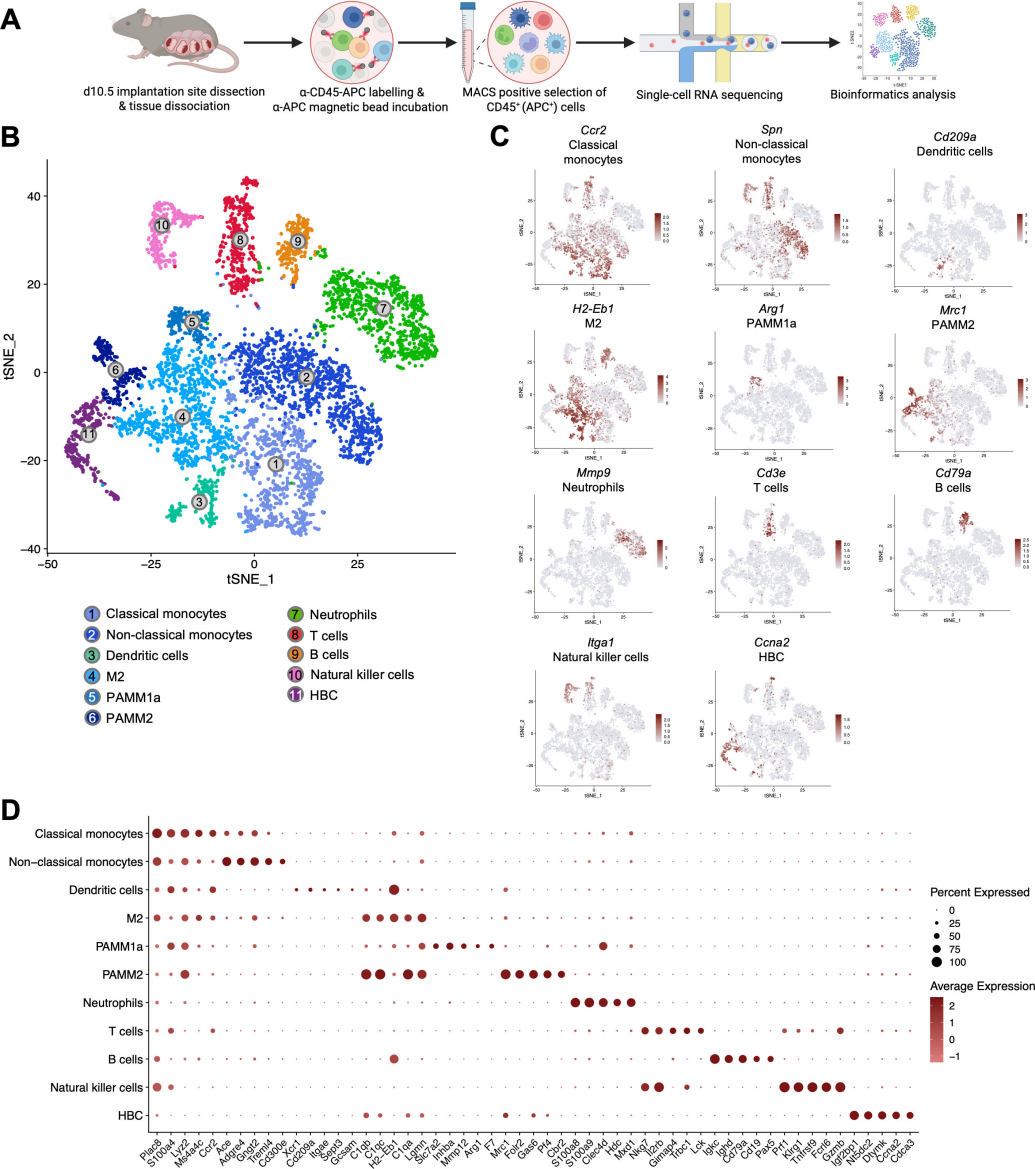
Identification of eleven distinct leukocyte populations by scRNA-seq. **(A)** Experimental workflow for the isolation and single-cell RNA-sequencing (scRNA-seq) analysis of cells at the d10.5 maternal-fetal interface. Four implantation sites from each female (Nodal^loxP/loxP^ n=4, Nodal^Δ/Δ^ n=4) were dissected, mechanically dissociated and enzymatically digested to generate single-cell suspensions. CD45^+^ leukocytes were enriched by magnetic activated cell sorting (MACS) prior to the pooling of two females (eight implantation sites) per sample (Nodal^loxP/loxP^ n=2, Nodal^Δ/Δ^ n=2), fixation and processing. Droplet-based encapsulation scRNA-seq was conducted (10X Genomics Chromium FLEX) and transcriptomic data analyzed. Created in BioRender. **(B)** t-SNE of the eleven distinct clusters of *Ptprc*^+^ cells identified through pseudo-bulk clustering. Clusters coloured according to cell type- blue; monocytes (1, 2) and macrophages (4–6), turquoise; dendritic cells (3), green; neutrophils (7), red; T cells (8), orange; B cells (9), pink; natural killer cells (10) and purple; Hofbauer cells (HBC, 11). **(C)** Representative t-SNE of marker genes used for the manual annotation of each cluster to cell type. **(D)** Dotplot of the top five highest-expressed marker genes (adjusted P-value<0.05) in each of the eleven immune cell clusters.

Leukocytes at the maternal-fetal interface reach a maximum abundance during placental development and have dual functions in the promotion of trophoblast invasion and spiral artery remodeling, while also preventing fetal rejection (5, 6, 38). This occurs between d10.5-d12.5 in the mouse or the late first trimester in humans and is a critical determinant for the progression of a healthy pregnancy (39–41). To investigate the transcriptomic profile of leukocytes during placentation and enhance the isolation of unique subpopulations, dissociated d10.5 myometrial, decidual and placental tissues from Nodal^loxP/loxP^ control and Nodal^Δ/Δ^ females were MACS-enriched for CD45^+^ cells prior to fixation, processing and scRNA-seq (10X Genomics Chromium FLEX) (**Figure 1A**). Following quality control and pseudo-bulk Seurat graph-based clustering of filtered *Ptprc*^+^ (CD45^+^) cells, eleven distinct clusters were identified from a total of 6,091 cells (**Figure 1B**). Clusters were annotated to cell types using the automatic ACT tool (42) and validated using known canonical markers (**Figure 1C**). Maternal leukocytes included two groups of monocytes; classical (*Ccr2*, *Sell*) and non-classical (*Spn*), three macrophage populations (*Mafb*, *Adgre1*, *Mpeg1*); M2 (*H2-Eb1*, *H2-Ab1*), PAMM1a (*Arg1, Inhba*) and PAMM2 (*Mrc1*, *Folr2*), and one cluster each of dendritic cells (*Cd209a*, *Cd74*, *Cst3*), neutrophils (*S100a9*, *S100a8*), T cells (*Cd3e*, *Lck*), B cells (*Cd19*, *Cd79a*, *Igkc*) and natural killer cells (*Nkg7*, *Itga1*, *Gzmb*). Fetal-derived placental macrophages (Hofbauer cells, HBCs) were also identified. The top 50 highest expressed marker genes (adjusted P-value<0.05) for each cluster are listed in **Supplementary Data 1**. Comparison of the top five marker genes in each cluster (**Figure 1D**) confirmed similarities between myeloid clusters, particularly M2 and PAMM2 cells, in addition to T cells and natural killer cells. Due to the identification of multiple myeloid cell subtypes and their current underrepresentation in the literature, emphasis was placed on characterizing the macrophage and neutrophil populations at the d10.5 maternal-fetal interface.

### Characterization of myeloid cells at the d10.5 maternal-fetal interface

#### Macrophages

Macrophages at the d10.5 maternal-fetal interface are a very heterogeneous population and are characterized by their compartment localization, cell origin and polarization state. Decidual macrophages originate from monocyte precursors and are a mixed M1/M2 or M2-predominant state at mid-gestation (43–45). Although it was presumed that maternal macrophages also resided within the placenta (PAMMs), this was only recently proven by the scRNA-seq analysis of human first trimester tissues and the d17.5 mouse placenta (20, 35). Conversely, fetal-origin macrophages or HBCs derived from the embryonic yolk sac have been described in the placental villus for decades (46–48) and are known to populate the mouse placenta by d10.5 (47).

**Figure 2:**
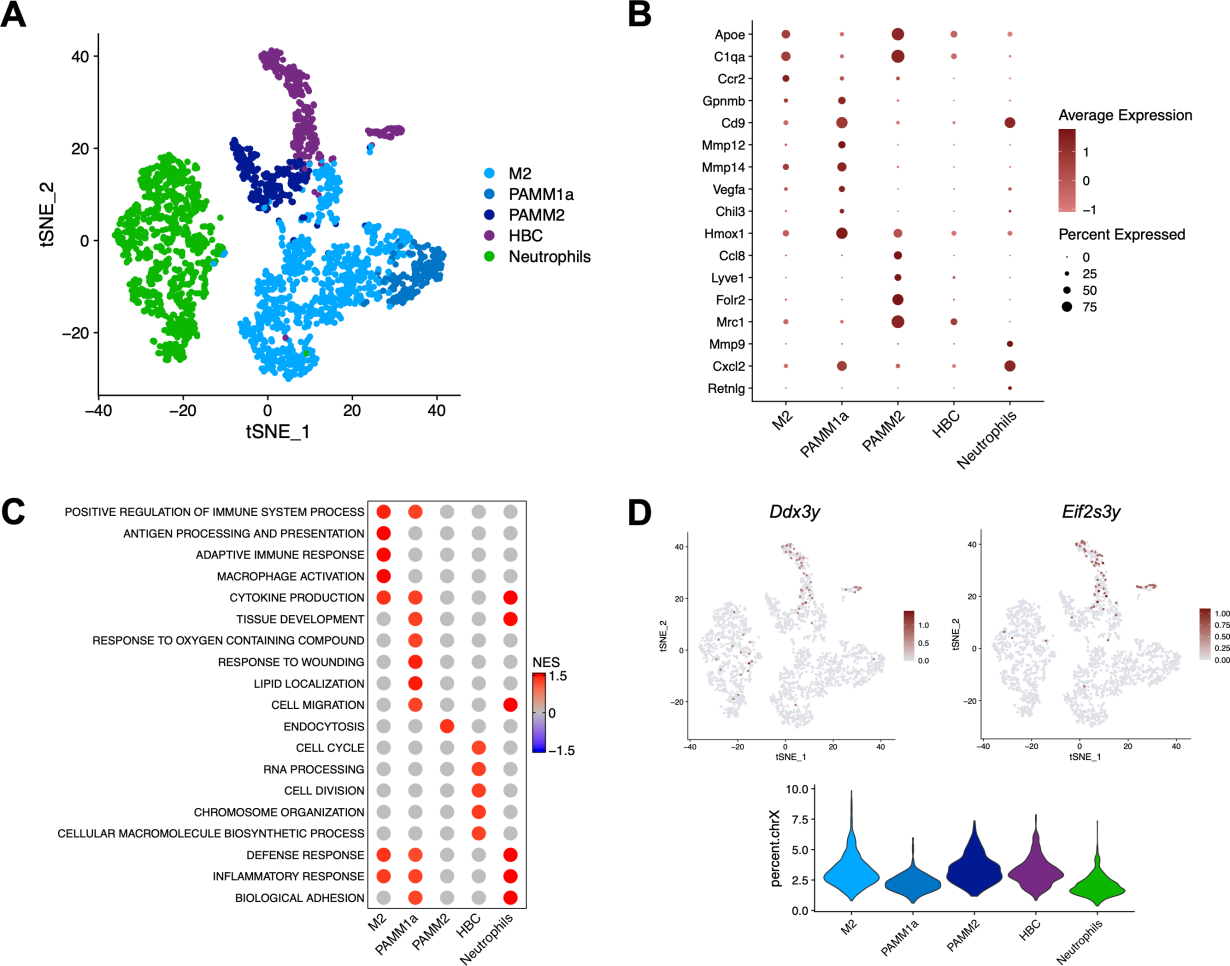
Macrophages and neutrophils in the d10.5 decidua and placenta. **(A)** t-SNE of the neutrophil and four macrophage clusters at the d10.5 maternal-fetal interface. **(B)** Dotplot expression of additional marker genes (adjusted P-value<0.05) for macrophage and neutrophil clusters at the d10.5 maternal-fetal interface. **(C)** Gene Ontology (GO) biological process terms for marker genes of macrophage and neutrophil populations (adjusted P-value<0.05). **(D)** t-SNE of the male-specific marker genes *Ddx3y* and *Eif2s3y* to distinguish between fetal HBCs and maternal macrophages on d10.5. Percentage of reads that mapped to the X chromosome showed female cells (maternal or fetal) in all clusters.

The most abundant macrophage population identified on d10.5 was the decidual M2 cluster (**Figure 2A**). M2 cells had an expression profile that aligned closely with M2-polarized anti-inflammatory macrophages from other scRNA-seq analyses of human first trimester (18, 19, 21) and mouse gestational tissues (36, 49). In addition to *C1qa/b/c*, *H2-Eb1* and *Lgmn* (**Figure 1D**), decidual M2 cells showed high expression of genes related to lipid metabolism and immune regulation such as *Acp5*, *Ctsb/d/l*, *Apoe*, *Lipa*, *Mafb*, *Lgals3*, *Fabp5*, *Trem2*, *Cxcl16*, *Mertk*, *Tgfbi*, *Selenop* and *Mmp14* (**Figure 2B**). These marker genes were associated with the Gene Ontology (GO) biological process terms “positive regulation of immune system processes”, “macrophage activation” and “antigen processing and presentation” (**Figure 2C**). The top 25 GO terms (adjusted P-value<0.05) for all cluster marker genes are listed in **Supplementary Data 2**.

The resolution of two additional maternal-origin macrophage clusters indicated the novel characterization of PAMMs in the d10.5 mouse placenta. The first cluster was labelled “PAMM1a” due to its close resemblance to human PAMM1a cells (20), as the expression of *Folr2* and *Ccr2* was absent but *Cd9* was highly enriched (**Figure 2B**). d10.5 PAMM1a cells were most similar to the d17.5 PAMM_Spp1 cluster (35). PAMM1a marker genes were associated with angiogenesis, lipid metabolism and immune regulation including *Arg1*, *Inhba* (**Figure 1D**), *Fn1*, *Orl1*, *Mmp8/12/13/14/19*, *Spp1*, *Vegfa*, *Lpl*, *Cd36*, *Ctsb/d/l*, *Lgmn*, *Lgals3*, *Cd63*, *Ilr7*, *Hmox1* and *Chil3* (**Figure 2B**). Moreover, PAMM1a cells replicated the enrichment of *Gpnmb* as described in other human equivalents (**Figure 2B**) (18, 19). Collectively, the properties of d10.5 PAMM1a cells were most reflected by the GO terms “tissue development”, “response to wounding” and “lipid localization” (**Figure 2C**), suggesting functions in tissue repair and macrophage phagocytosis.

The second PAMM cluster was defined as “PAMM2” since it matched the FOLR2^+^ CD9^-^ CCR2^-^ PAMM2 population identified by flow cytometry of human first trimester samples (20). Previously considered analogous to decidual macrophages (20), here d10.5 mouse PAMM2 cells formed a similar but distinct cluster from decidual M2 cells in scRNA-seq (**Figures 1B**, **2A**). In addition to the enrichment of *Folr2* and absence of *Cd9* and *Ccr2* (**Figure 2B**), PAMM2 cells were recognized by the high expression of *Mrc1*, *Pf4* (**Figures 1D**, **2B**), *Lyve1*, *Ccl8*, *C1qa/b/c*, *Apoe* (**Figure 2B**), *Fcna*, *C4b* and *Dab2*. PAMM2 cells were also comparable to the d17.5 PAMM_Ccl8 population (35). “Endocytosis” was the singular marker GO term affiliated with PAMM2 cells (**Figure 2C**), which may indicate a role for this population in placental development and nutrient transport.

The evaluation of male-specific *Ddx3y* and *Eif2s3y* gene expression was used to overcome the challenge of defining HBCs as a separate population from maternal-origin M2, PAMM1a and PAMM2 cells. Due to the random inclusion placentae from male and female embryos, the fetal HBC cluster showed enrichment for the two male-specific genes (**Figure 2D**). The use of male-specific markers was most important to define PAMM2 cells, as the expression of *Mrc1*, *Folr2* and *Lyve1* is typically associated with human HBCs (18, 20, 35). Since HBCs in mice and humans differ in cell ontology and localization within the placenta (50), and *Folr2* was previously reported to be an inadequate marker for mouse d17.5 HBCs (35), it is now proposed that *Mrc1*, *Folr2* and *Lyve1* are also insufficient markers for mid-gestational mouse HBCs. Instead, as HBCs enter the placenta by d10.5 (47), they resemble proliferating and primitive macrophages with marker GO terms exclusively related to cell division and cycling (**Figure 2C**). Therefore, although the characterization of fetal HBCs was outside the scope of this study, it is reiterated that male-specific markers are required to determine the true origin of macrophages present at the maternal-fetal interface.

#### Neutrophils

Neutrophils exhibit functional plasticity throughout pregnancy that counteracts their more well-defined pro-inflammatory role in phagocytosis, cytotoxic granule production and neutrophil extracellular trap (NET) formation. Instead, decidual neutrophils are polarized towards an immunosuppressive and pro-angiogenic state, mirroring the M1/M2 dichotomy and producing factors to assist in tissue remodeling and vascularization (51). On d10.5 during normal placental development, neutrophil marker genes that reflected these pregnancy-associated functions included *Mmp9*, *Cxcl2*, *Retnlg* (**Figure 2B**), *Mmp8*, *Wfdc21*, *Cxcr2*, *Csf3r* and *Junb*. These genes were associated with the GO biological process terms “defense response”, “inflammatory response” and “cell migration” (**Figure 2C**).

### Changes in the abundance and gene expression profile of Nodal^Δ/Δ^ leukocytes

**Figure 3:**
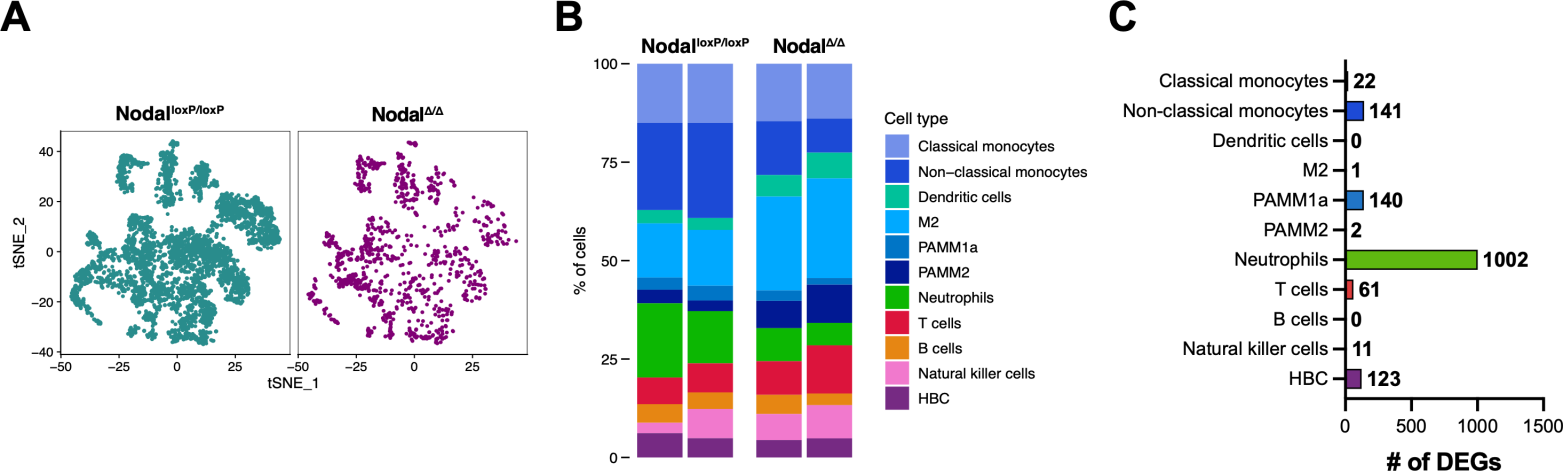
Changes in the abundance and gene expression profile of leukocytes in Nodal^Δ/Δ^ females. **(A)** t-SNE of all *Ptprc*^+^ cells at the Nodal^loxP/loxP^ (n=2) and Nodal^Δ/Δ^ (n=2) maternal-fetal interface. **(B)** Proportion of cell types in each sample. Notably, the proportion of maternal neutrophils, non-classical monocytes and PAMM1a cells was significantly decreased in Nodal^Δ/Δ^ females, while the proportion of M2 and PAMM2 cells was increased (FDR<0.05). **(C)** The number of significant differentially expressed genes (DEGs) (adjusted P-value<0.1) in all immune cell clusters at the d10.5 maternal-fetal interface between Nodal^loxP/loxP^ and Nodal^Δ/Δ^ females.

The reproductive phenotypes described in the Nodal^Δ/Δ^ model (11, 14) provided a unique opportunity to investigate maternal immune dysregulation during placental development and how it was associated with pregnancy complications like fetal loss, IUGR and PE. A total of 4,938 cells from Nodal^loxP/loxP^ females and 1,153 cells from Nodal^Δ/Δ^ females were used for differential analysis (**Figure 3A**). The most notable changes in leukocyte abundance between groups was observed in myeloid cells, as Nodal^Δ/Δ^ females had a significant decrease in the proportion of neutrophils (Nodal^loxP/loxP^ 16.1%, Nodal^Δ/Δ^ 7.1% of leukocytes), non-classical monocytes (Nodal^loxP/loxP^ 23.2%, Nodal^Δ/Δ^ 11.2% of leukocytes) and PAMM1a cells (Nodal^loxP/loxP^ 3.4%, Nodal^Δ/Δ^ 2.2% of leukocytes). In Nodal^Δ/Δ^ females there was an almost two-fold increase in the proportion of M2 (Nodal^loxP/loxP^ 14.0%, Nodal^Δ/Δ^ 24.6% of leukocytes) and PAMM2 cells (Nodal^loxP/loxP^ 3.1%, Nodal^Δ/Δ^ 8.3% of leukocytes). Flow cytometry validated the reduction of neutrophils in Nodal^Δ/Δ^ females (**Supplementary Figure 1**) but was not able to identify monocyte and macrophage subpopulations analogous to those in scRNA-seq due to the simplicity of markers used. Furthermore, the decrease in the number of Nodal^Δ/Δ^ neutrophils was only observed directly at the d10.5 maternal-fetal interface and not systemically, as the composition of matched peripheral blood (**Supplementary Figures 2A, B**) and splenic (**Supplementary Figures 2C, D**) leukocytes remained unchanged. The proportion of lymphocytes was also the same between groups (**Figure 3B**; **Supplementary Figure 1**, **Supplementary Figure 2**).

Differentially expressed genes (DEGs) were then assessed to identify changes in leukocyte gene expression profile in the context of Nodal^Δ/Δ^ reproductive pathologies. The most pronounced difference in Nodal^Δ/Δ^ females compared to Nodal^loxP/loxP^ controls was observed in the neutrophils with 1002 DEGs. Additionally, despite the small population size, the Nodal^Δ/Δ^ PAMM1a population had 141 DEGs (**Figure 3C**). The top 50 DEGs (adjusted P-value<0.1) for each cluster are listed in Supplementary Data 3 and displayed as a heatmap in **Supplementary Figure 3**. Since the role of PAMM1a cells and neutrophils in reproductive failure is unknown or poorly defined, these two populations were characterized in Nodal^Δ/Δ^ females.

### Differentially expressed genes and gene ontology analysis of Nodal^Δ/Δ^ PAMM1a cells and neutrophils

**Figure 4:**
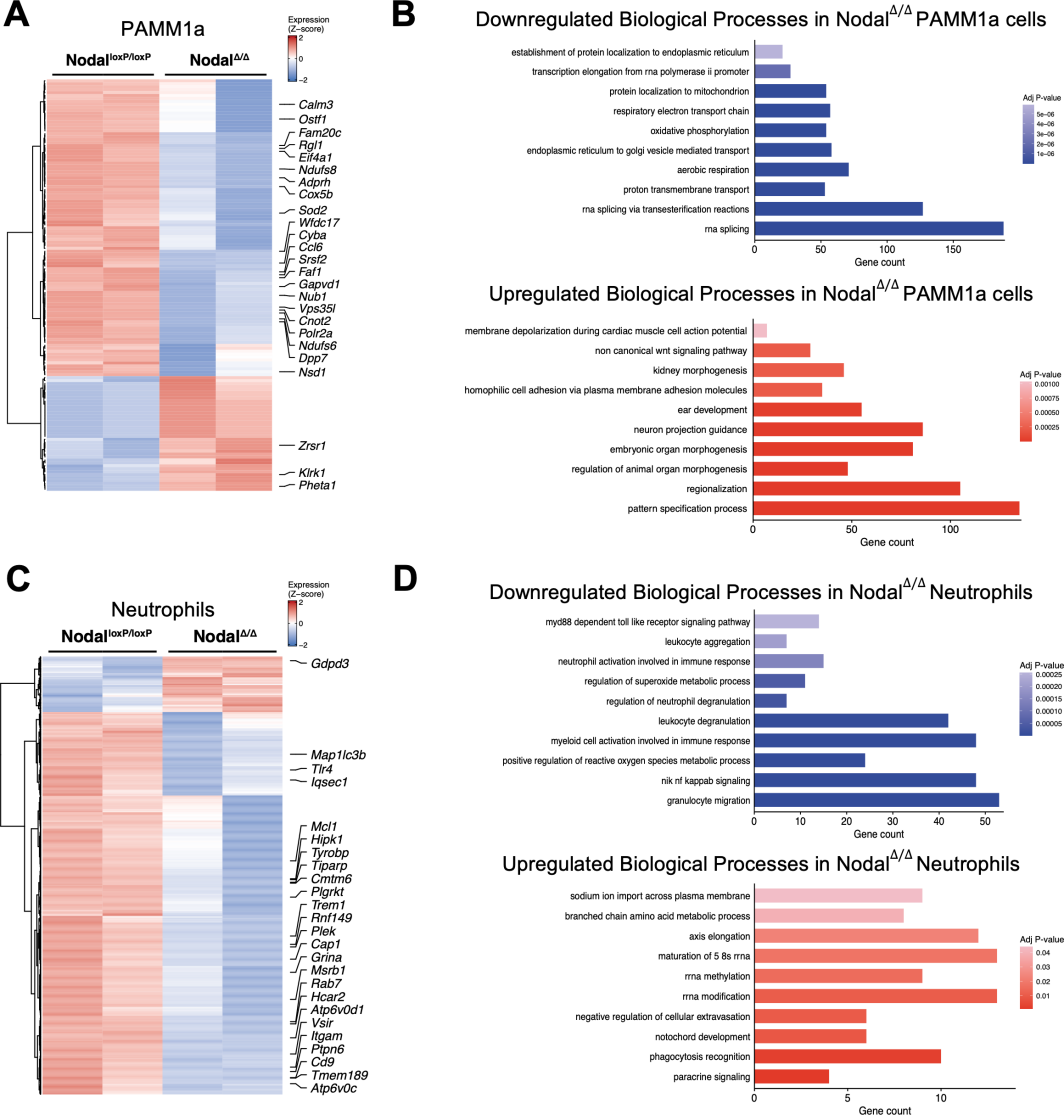
Differentially expressed genes and Gene Ontology analysis of Nodal^Δ/Δ^ PAMM1a cells and neutrophils. **(A)** Heatmap of significant differentially expressed genes (DEGs) (adjusted P-value<0.1) between Nodal^loxP/loxP^ (n=2) and Nodal^Δ/Δ^ (n=2) females in PAMM1a cells. The top 25 upregulated or downregulated DEGs are labelled. **(B)** The top 10 downregulated or upregulated Gene Ontology (GO) biological process terms of Nodal^Δ/Δ^ PAMM1a DEGs (adjusted P-value<0.05, ranked by NES). **(C)** Heatmap of significant DEGs between Nodal^loxP/loxP^ and Nodal^Δ/Δ^ females in neutrophils. The top 25 upregulated or downregulated DEGs are labelled. **(D)** The top 10 downregulated or upregulated GO biological process terms of Nodal^Δ/Δ^ neutrophil DEGs.

To understand how PAMM1a cells and neutrophils differ in the context of reproductive failure, analysis of specific DEGs and their associated GO biological process terms was conducted. Nodal^Δ/Δ^ PAMM1a cells had 101 genes significantly downregulated and 39 upregulated, including the decreased expression of many angiogenic and immunosuppressive genes such as *Cnot2*, *Ndufs8*, *Gapvd1*, *Wfdc17*, *Ccl6* and *Cyba* (**Figure 4A**). These downregulated DEGs were associated with GO biological process terms “oxidative phosphorylation” and “aerobic respiration” (**Figure 4B**). Upregulated PAMM1a DEG GO terms included developmental processes like “pattern specification” and “regionalization” (**Figure 4B**).

In the neutrophils of Nodal^Δ/Δ^ females, 875 genes were downregulated and 127 upregulated compared to Nodal^loxP/loxP^ controls. Many of these downregulated DEGs were related to pro-inflammatory neutrophil processes like *Trem1*, *Tlr4*, *Rab7*, *Msrb1*, *Map1lc3b* and *Atp6v0d1* (**Figure 4C**), and reflected by the downregulated GO terms “neutrophil activation involved in immune response”, “regulation of neutrophil degranulation” and “positive regulation of reactive oxygen species metabolic process”. Upregulated neutrophil GO terms were involved in RNA and protein synthesis like “rRNA methylation” and “branched chain amino acid metabolic process” (**Figure 4D**). A list of the top 25 upregulated and downregulated DEG GO terms is included in Supplementary Data 4. Therefore, in the context of the Nodal^Δ/Δ^ model, the transcriptional profile of PAMM1a cells suggested reduced angiogenic and immunosuppressive functions and neutrophil signatures consistent with decreased pro-inflammatory activity.

### Elevated pro-inflammatory gene expression at the Nodal^Δ/Δ^ maternal-fetal interface

**Figure 5:**
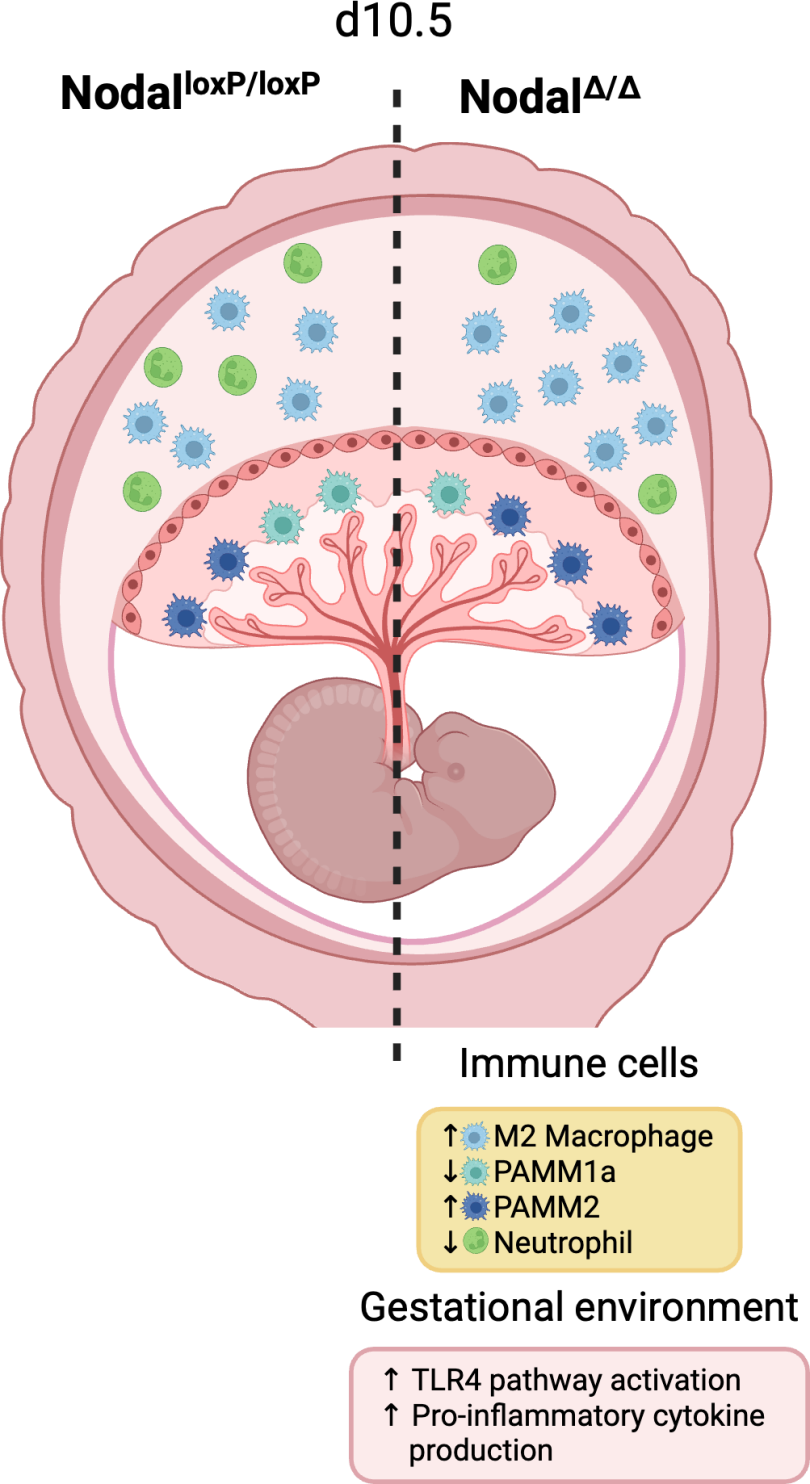
Altered myeloid cell abundance and pro-inflammatory cytokine expression at the d10.5 Nodal^Δ/Δ^ maternal-fetal interface. Nodal^Δ/Δ^ females show a reduced proportion of PAMM1a cells and neutrophils, and an increased abundance of M2 and PAMM2 cells at the d10.5 maternal-fetal interface. Collectively, the Nodal^Δ/Δ^ gestational environment is increased in TLR4 pathway activation and pro-inflammatory cytokine expression in comparison to Nodal^loxP/loxP^ controls. Created in BioRender.

In recognition that leukocytes do not exclusively influence the gestational inflammatory environment and pregnancy outcome, the relative expression of select genes involved in innate and adaptive immune responses was assessed in all cells of the d10.5 myometrium, decidua and placenta. Bulk mRNA was isolated from Nodal^Δ/Δ^ and Nodal^loxP/loxP^ females and 34 genes were found to be significantly upregulated in Nodal^Δ/Δ^ females by RT-qPCR profiler array (**Supplementary Table 1**). Notably, both branches of the TLR4 inflammatory pathway were activated in Nodal^Δ/Δ^ females, as shown by increased expression of the *Tlr4*, *Cd14* and *Md2* receptor complex, the MYD88-dependent signaling branch (*Irak1*, *Mapk1*, *Mapk8*, *Nfkbia* and *Nfkb1*) and the MYD88-independent branch (*Irf3*). Downstream products of TLR4 signaling were also increased in Nodal^Δ/Δ^ females as demonstrated by the high expression of pro-inflammatory cytokines *Il6*, *Il1a*, *Il2*, *Il18*, *Il1b* and interferon induced *Cxcl10*. Taken together, it is predicted that increased TLR4 pathway activation and pro-inflammatory cytokine production in the Nodal^Δ/Δ^ gestational environment directs the abundance and function of leukocytes at the maternal-fetal interface (**Figure 5**).

## Discussion

Due to the rarity of human first trimester samples, the necessity of a mouse model that can reflect immune-mediated reproductive failure has become increasingly relevant. While it is recognized that the timing of decidualization, location and depth of spiral artery remodeling and uNK phenotype differs between species (6, 38, 52), studies of mouse models can elevate findings and identify mechanisms of pregnancy regulation otherwise clinically and ethically impossible to discern from human studies alone. Since 50% of Nodal^Δ/Δ^ females establish a pregnancy despite an excess of inflammatory macrophages and lack of FOXP3^+^ Tregs during the preimplantation period (11), it is expected that this impaired maternal immune environment serves in part as an upstream driver of the placental insufficiency, fetal loss, IUGR and PE-like phenotypes observed at mid-gestation in the Nodal^Δ/Δ^ model (11, 14, 15), reinforcing recent hypotheses from corresponding human reproductive pathologies (1, 17, 53).

Here, the use of PAMM nomenclature derived human first trimester tissues was applied for the first time to the d10.5 mouse maternal-fetal interface. In humans, PAMM1a cells adhere to the placental syncytium and are localized to sites of tissue damage as breaks in the placental barrier and fibrin deposits are a common feature of physiological pregnancy (20, 54). Although there are some structural differences in the murine labyrinth and human villous placenta, d10.5 mouse PAMM1a cells are expected to inhabit the placental labyrinth layer through the connection between the maternal and fetal circulation. The production of MMP9 and expression of *Fn1* in human PAMM1a cells indicates functions in tissue regeneration and repair, while the presence of lipid droplets is related to macrophage phagocytosis (20, 55). d10.5 PAMM1a cells were proven to be analogous to human PAMM1a cells by the enriched expression of various *Mmp*s, *Fn1*, *Lgals3*, *Gpnmb*, *Lpl*, *Lipa*, *Lgmn* and *Fabp5* (**Figure 2B**), and by the GO biological process terms related to “wound healing” and “lipid metabolism” (**Figure 2C**).

In Nodal^Δ/Δ^ females, the decreased abundance of PAMM1a cells (**Figure 3B**) suggested a reduced capacity for repair of the placental barrier, which could leave the labyrinth more vulnerable to stress and the accumulation of inflammatory maternal leukocytes. Increased breaks and fibrin deposits within the placenta is frequently described in PE (56–58), while excess villus inflammation is highly associated with IUGR and an increased risk for pregnancy loss (59, 60). In some cases these conditions are a consequence of failed maternal tolerance (1, 38, 59, 61–63), which is reiterated by phenotypes in the Nodal^Δ/Δ^ model (11, 14). Of all maternal macrophage populations, PAMM1a cells showed the highest expression of *Vegfa* and *Arg1* (**Figure 2B**), both well characterized angiogenic and anti-inflammatory factors (7, 64–69). However, many of the downregulated genes identified in PAMM1a cells of Nodal^Δ/Δ^ females were positive regulators of angiogenesis, VEGF signaling and M2 polarization (**Figure 4A**). For example, *Cnot2* and *Ndufs8* expression in endothelial cells enhances proliferation and capillary tube formation (70, 71), while *Gapvd1* is expressed in response to macrophage-derived VEGF in tumors (72). *Wfdc17*, *Ccl6* and *Cyba* are associated with the immunosuppressive and M2 polarization state of macrophages (73–77). This was further confirmed by the downregulated biological process terms “oxidative phosphorylation” and “aerobic respiration” in Nodal^Δ/Δ^ PAMM1a cells (**Figure 4B**), since these are processes required to maintain anti-inflammatory macrophage polarization (78, 79). Therefore, PAMM1a cells not only contribute to the repair of the placental barrier but also have expression profiles indicative of the regulation of angiogenesis and immune suppression, with the potential to support healthy placental development.

An additional subset of PAMM cells called “PAMM1b” was previously described in human first trimester tissues (20), however it was not resolved as a separate cluster in the mouse d10.5 placenta. Since PAMM1b cells are monocyte precursors to PAMM1a cells (20), shared properties with the d10.5 classical monocyte cluster could explain the absence of a specific PAMM1b population in this dataset. Conversely, d10.5 PAMM2 cells formed a similar but distinct cluster from decidual M2 cells (**Figures 1B**, **2A**). This revealed a potential placental niche-specific role for PAMM2 cells in endocytosis (**Figure 2C**). Although the precise mechanism of endocytosis at the placental barrier remains elusive, it is proposed that PAMM2 macrophages assist in the transport of nutrients, hormones and other proteins from the maternal circulation to nearby trophoblast cells of the fetal villus during placentation (80). Surprisingly, despite the near doubling of PAMM2 and M2 cells in Nodal^Δ/Δ^ females (**Figure 3B**), the number of significant DEGs between groups was very minimal (**Figure 3C**). Instead, it is predicted that the recruitment, differentiation or maintenance of these leukocyte populations was affected in Nodal^Δ/Δ^ females rather than their transcriptional profile.

The evaluation of neutrophils in physiological pregnancy is underrepresented in current literature. The traditional association of neutrophil activation with pro-inflammatory functions like phagocytosis and NET formation is challenged in pregnancy by the shift towards a more immunosuppressive and angiogenic neutrophil profile (51, 81–85). The regulation of this state is important as the transition back to pro-inflammatory activation can contribute to reproductive complications (51, 85). Here, marker genes of d10.5 decidual neutrophils confirmed an anti-inflammatory profile, and it was noted that neutrophils had the highest *Mmp9* expression of all myeloid cells (**Figures 1C**, **2B**). Intriguingly, although many DEGs were identified in Nodal^Δ/Δ^ neutrophils (**Figure 3C**), most were associated with the downregulation of pro-inflammatory processes expected in physiological pregnancy. For example, *Trem1* amplifies inflammatory pathways triggered by *Tlr4* and the production of pro-inflammatory cytokines (86–88), *Atp6v0d1* is involved in neutrophil antigen presentation (89) and *Msrb1* is a potent anti-oxidant that protects against reactive oxygen species release during degranulation (90). Moreover, *Map1lc3b* promotes autophagy and NET formation (91), while *Rab7* is involved in early phagosome assembly (92). This lack of inflammatory neutrophil response was reflected in the downregulated GO terms “neutrophil activation involved in immune response”, “regulation of neutrophil degranulation” and “regulation of superoxide metabolic processes” (**Figure 4D**). The number of decidual neutrophils was also dramatically reduced by 50% in Nodal^Δ/Δ^ females (**Figure 3B**; **Supplementary Figure 1C**) and further highlighted by the downregulated GO term “granulocyte migration” (**Figure 4D**). Together, these findings contrasted with other studies that reported an increase in neutrophil activation and frequency during pregnancy loss or PE (51, 93–95). Two possibilities could further explain this phenomenon in the Nodal^Δ/Δ^ model. First, a decrease in neutrophil abundance could represent the loss of beneficial angiogenic and immunosuppressive cells during placental development, presumably before the switch to increased local neutrophil activation. Alternatively, it could demonstrate the loss of neutrophil phagocytic capabilities that are important in a dysfunctional placental environment. This neutrophil “immunoregulatory confusion” was previously demonstrated in PE, where the removal of excess trophoblast-derived debris under hypoxic conditions was less efficient due to defective neutrophil phagocytosis (84). Both hypotheses of neutrophil dysregulation could contribute to the reproductive phenotypes observed in the Nodal^Δ/Δ^ model.

It is important to acknowledge that the differentiation of myeloid cells occurs in response to the local inflammatory environment and is thus dependent on signaling cues from other tissue-resident cells at the maternal-fetal interface (96–98). Secreted factors from other leukocyte populations (**Supplementary Figure 3**) and non-immune cell types like decidual stromal cells, endothelial cells and fetal trophoblasts considerably influence the inflammatory environment during placental development (8, 99–101). Collectively, the d10.5 Nodal^Δ/Δ^ maternal-fetal interface was pro-inflammatory (**Figure 5**; **Supplementary Table 1**), which is consistent with pregnancy loss, IUGR and PE in humans or other related murine models (65–67, 102–113). However, it is still unknown if this excess inflammation was a downstream effect of earlier preimplantation immune dysregulation (11), the consequence of impaired angiogenesis and trophoblast differentiation (15), or compensatory adaptations which led to placental insufficiency and reproductive failure. Therefore, future studies should aim to completely define how both immune and non-immune populations change throughout gestation in physiological and pathological pregnancies.

uNKs are the most abundant leukocytes during placentation and are often the focus of studies in reproduction (4, 6, 114, 115). *Dolichos biflorus* agglutinin (DBA) lectin is a common marker of mouse uNKs (116, 117), and DBA^+^ uNKs are numerous within d10.5 Nodal^loxP/loxP^ and Nodal^Δ/Δ^ implantation sites by immunofluorescence (**Supplementary Figures 4A, B**). Surprisingly, the majority of DBA^+^ uNKs were CD45^-^ on d10.5 (**Supplementary Figure 4B’–D**), and the minor CD45^+^ natural killer cell population identified (**Figure 1B**; **Supplementary Figure 1**) was more analogous to conventional natural killer cells (18). These findings were validated in a separate study, where spatial transcriptomics of the d10.5 maternal-fetal interface showed only 7% of decidual natural killer cells as *Ptprc*^+^ (CD45^+^) (118). Therefore, due to MACS positive selection, the major CD45^-^ DBA^+^ population of uNKs was not included in the scRNA-seq dataset and is acknowledged as a limitation of this study, which should be considered when interpreting the complete immune landscape.

The total number of leukocytes isolated from Nodal^Δ/Δ^ females for scRNA-seq was approximately 75% less than from Nodal^loxP/loxP^ females (**Figure 3A**). A smaller d10.5 Nodal^Δ/Δ^ decidua with more cell apoptosis and less proliferation (14) is the probable cause for discrepancies in total leukocyte number, which could complicate the feasibility of future differential analysis of lymphocyte subpopulations and underestimate proportional variability between groups.

In conclusion, the single-cell characterization of maternal myeloid populations within the d10.5 decidua and placenta provided insights into leukocyte biology directly at the maternal-fetal interface in physiological and pathological Nodal^Δ/Δ^ pregnancies. In particular, the resolution of PAMM1a and PAMM2 cells in the mouse d10.5 placenta suggested novel roles for these previously uncharacterized populations during placental development. Ultimately, the balance between myeloid cell abundance and their expression profile during placentation is a critical determinant of reproductive outcome and pregnancy success.

## Materials and methods

### Generation and maintenance of Nodal deficient mice

The generation of these mice has been previously described (14). Mice with loxP sites flanking exons 2 and 3 of the *Nodal* gene (*Nodal*^loxP/loxP^) on a mixed background were kindly donated by E.J. Robertson (University of Oxford, UK) (119). Progesterone receptor (*Pgr)*-Cre females (*Pgr^Cre^*^/+^) on a C57BL6/129 background were generously provided by F.J. DeMayo and J.P. Lydon (Baylor College of Medicine, USA) (120). Both strains have been previously reported to demonstrate normal fertility. *Nodal*^loxP/loxP^ and *Pgr*^Cre/+^ strains were crossed, and the offspring were genotyped by PCR. In this study, 8–12-week-old *Nodal* floxed control (*Nodal*^loxP/loxP^/*Pgr*^+/+^ - simplified as Nodal^loxP/loxP^) and *Nodal*-deficient (*Nodal*^loxP/loxP^/*Pgr*^Cre/+^ - Nodal^Δ/Δ^) virgin females were used as experimental mice. Females were mated with a wild-type CD1 male overnight and the presence of a copulatory plug the following morning indicated successful mating and day 0.5 (d0.5) of pregnancy.

### Magnetic activated cell sorting

Four implantation sites were isolated from each female on d10.5 of pregnancy. Following dissection and removal of the embryos, the remaining maternal decidua, myometrium and fetal placenta tissues (from both male and female embryos) were pooled and collectively referred to as the “maternal-fetal interface”. Both mechanical dissociation and enzymatic digestion were used to generate a single-cell suspension from fresh tissues as previously described (11). Briefly, cells were digested with Liberase TM (Roche Cat. No. 5401119001) in HBSS supplemented with 2% FBS (25 μg Liberase per 0.1 g of tissue) for 45 minutes at 37 °C, with agitation every 15 minutes. Red blood cells were removed by incubation in 5 mL ACK lysis buffer pH 7.2 (150 mm/L NH_4_Cl, 10 mm/L KHCO_3_, 0.1 mm/L Na_2_EDTA) for 2 minutes. Products were filtered through a 70 μm cell strainer. Cells were stained with anti-mouse CD45-APC (BioLegend Cat. No. 103112) for 30 minutes and then incubated with anti-APC microbeads (Miltenyi Biotec Cat. No. 130-090-855) for 15 minutes following manufacturer’s protocol. APC^+^ cells (CD45^+^ leukocytes) were positively selected for using the Miltenyi Biotec AutoMACS Pro Separator at the Immunophenotyping Platform of the Research Institute of the McGill University Health Centre (RI-MUHC).

### Single-cell library preparation and RNA-sequencing

Following MACS separation, the positively selected CD45^+^ leukocytes were pooled such that one sample represented eight implantation sites from two females of the same group (Nodal^loxP/loxP^ or Nodal^Δ/Δ^). Cells were fixed and processed using the Chromium Next GEM Single Cell Fixed RNA Sample Preparation Kit (10X Genomics Cat. No. PN-1000414) according to protocol #CG000478. Probe hybridization was performed on approximately 500,000 cells/sample using the Chromium Fixed RNA Mouse Transcriptome Kit (10X Genomics Cat. No. PN-1000497) following protocol #CG000527. 18,000 cells were encapsulated for a target library of 10,000 cells/sample on the 10X Genomics Chromium iX, prepared by the Genomics Platform at the Research Centre of the CHU Sainte-Justine. Library quality control and single-cell RNA-sequencing (scRNA-seq) was conducted on the Illumina NovaSeq6000 S4 v1.5 PE100 at the McGill Genome Centre.

### Single-cell RNA-sequencing data processing and analysis

Droplet libraries were first processed using the Cell Ranger multi pipeline (121). Sequencing reads were aligned to the mm10 mouse reference genome, and transcript counts quantified for each annotated gene within every cell. Count matrices (genes × cells) were loaded into the R package Seurat (122) for quality control and downstream analyses. *Ptprc*^+^ cells (read count ≥1) were selected and low-quality cells were subsequently filtered out using the following criteria: the number of detected genes ≤ 300, percentage of mitochondrial RNA ≥ 10% per cell. Cell doublets were detected and removed using the R package scDblFinder (123). Following SCTransform normalization, individual samples were integrated using the Harmony procedure. The RunTSNE function was used to compute a t-SNE dimensional reduction based on the first 20 principal components. A nearest-neighbor graph using the first 20 principal components was calculated using FindNeighbors function, followed by clustering using FindClusters function with resolution =0.4. Cluster-specific marker genes were identified using the function FindMarkers with cutoffs: log2 fold-change >0.5 and adjusted P-value<0.05 (upregulated genes only). Clusters were annotated to cell types by inputting the top 50 highest-expressed genes into the automatic cell type annotation tool ACT (42), followed by manual validation based on canonical markers. Differential composition analysis was performed using the R package sccomp (124). Per-cluster differential expression testing between two groups was conducted using a pseudo-bulk approach. This was accomplished by summing counts together for all cells with the same combination of sample and cluster. Lowly-expressed genes were excluded with an average read count lower than 10 across all samples in each cluster. Raw counts were normalized using edgeR’s TMM with singleton pairing algorithm (125) and were then transformed to log2-counts per million (log2CPM) using the voomLmFit function implemented in the R package limma (126). Gene set enrichment analysis based on pre-ranked marker gene list by t-statistic was performed using the R package fgsea (127) and rrvgo (128) to simplify the redundancy of Gene Ontology (GO) biological process terms based on semantic similarity. To assess differences in gene expression levels, a linear model was fit using the lmfit function considering batch effects. Nominal p-values were corrected for multiple testing using the Benjamini-Hochberg method. Significant differentially expressed genes were defined using a P-adjusted value threshold of 0.1. Single-cell RNA-sequencing data processing and analysis was conducted by the Canadian Centre for Computational Genomics at the Victor Phillip Dahdaleh Institute of Genomic Medicine at McGill University.

### Flow cytometry

Implantation sites were isolated and processed as described for MACS. To generate a single-cell suspension from the spleen, tissues were pushed through a cell strainer with a syringe plug and treated with ACK buffer for 30 seconds. Matched blood samples were isolated from d10.5 females by cardiac puncture and were treated twice with ACK lysis buffer directly. To facilitate intracellular cytokine staining, cells were stimulated with Cell Activation Cocktail (phorbol-12-myristate 13-acetate and ionomycin, BioLegend Cat. No. 423301) following manufacturer’s protocol for 4 hours in HBSS/2% FBS. Monensin (BioLegend Cat. No. 420701) transport inhibitor was added during the last hour of incubation. 1 million cells/sample were blocked using the FcγR antibody for 10 minutes (BD Biosciences Cat. No. 553142). The panel of fluorophore-conjugated antibodies used to identify immune cell populations residing at the maternal-fetal interface is listed in **Supplementary Table 2**, T cell subpopulations in **Supplementary Table 3**, and within the blood and spleen in **Supplementary Table 4**. Samples were stained with extracellular markers for 30 minutes and fixed overnight using the FoxP3/Transcription Factor Staining Buffer Set (Invitrogen Cat. No. 00-5523-00). After permeabilization, cells were stained with intracellular markers for 30 minutes as necessary. Compensation was performed using UltraComp eBeads (Invitrogen Cat. No. 01-2222-42). All FMO controls were used during panel validation and again as needed for CD11c and F4/80. An unstimulated control was used during cell activation for intracellular cytokine staining. Samples were processed on the LSRFortessa X-20 (BD Biosciences) at the Immunophenotyping Platform of the RI-MUHC. Flow cytometry data was analyzed by the FlowJo software (BD Biosciences Version 10.8.1). Statistical analysis was performed first by the removal of outliers (ROUT, Q = 1%) and then two-tailed unpaired Student t-tests using GraphPad Prism (Version 10.0.3). P-values<0.05 were considered statistically significant.

### RT^2^ quantitative PCR profiler array

Four implantation sites were isolated from each female on d10.5 as described and stored at -80 °C until use. Samples were homogenized and total RNA extracted using Trizol (Invitrogen Cat. No. 15596018) and the RNeasy Mini Kit (Qiagen Cat. No. 74104). The RT^2^ First Strand Kit (Qiagen Cat. No. 330401) was used for cDNA synthesis. Reverse transcription quantitative PCR (RT-qPCR) was performed using the RT^2^ SYBR Green qPCR Mastermix (Qiagen Cat. No. 330500), and the Mouse Innate and Adaptive Immune Responses (PAMM-052Z) profiler array (Qiagen Cat. No. 330231) following manufacturer’s protocol. The 96-well array contained 84 genes of interest, five housekeeping genes, one internal control for genomic DNA contamination, three reverse transcription controls for assessment of RNA quality and three positive controls for general determination of PCR performance. Samples were run on the Roche LightCycler 480 thermocycler and analyzed using Qiagen GeneGlobe software. Relative expression of the gene of interest was calculated by the ΔΔCt method, where average fold change in Nodal^Δ/Δ^ gene expression was relative to Nodal^loxP/loxP^ mice and normalized to the five housekeeping genes (*Actb, B2m, Gapdh, Gusb* and *Hsp90ab1*). Statistical analysis was performed by Student’s t-tests, p-values<0.05 were considered statistically significant.

### Tissue processing, paraffin embedding and sectioning

Paraffin embedding and tissue histology methods were employed as previously described (11, 129). Briefly, the entire d10.5 uterus was collected in HBSS and dissected into sets of three or four implantation sites (including the embryo) before fixation in 10% neutral buffered formalin for a minimum of 48 hours at 4 °C. Samples were dehydrated in increasing concentrations of ethanol and cleared in xylene before embedded into paraffin wax (Tissue Tek). Blocks were slowly solidified on a cold plate for one hour before transfer to a -20 °C freezer until sectioning. 7 μm transverse sections were cut using the RM2145 microtome (Leica) and mounted onto Superfrost Plus (Fisher) slides.

### Immunofluorescence

Immunofluorescence staining was conducted as previously described (11, 129). Briefly, slides were deparaffinized in xylene and rehydrated in a decreasing ethanol gradient. Antigen retrieval was conducted in 10 mM sodium citrate with 0.05% Tween 20, pH 6 at 95 °C for 20 minutes. Slides were cooled and permeabilized in TBT (TBS, 2.5% TritonX-100) for 15 minutes. Slides were blocked for an hour at room temperature in 10% BSA and then washed with TBST (TBS, 0.025% Tween 20). Slides were incubated with CD45 primary antibody (BioLegend Cat. No. 103102, 1:100) overnight at 4 °C. The following day, slides were washed in TBST and incubated for 2 hours at room temperature with the secondary antibody donkey α-rat Alexa Fluor 594 (Invitrogen Cat. No. A21209, 1:300), DBA-FITC (Bio World Cat. No. 21761015-1, 1:300) and DAPI (ThermoScientific Cat. No. 62248, 1:500). Slides were washed and mounted with Mowiol 4-88 (Calbiochem). Slides were imaged with the DMI6000 inverted epifluorescence microscope (Leica). Images were reconstructed in Adobe Photoshop CS6 (Version 13.0) using the photo-merge tool.

## Data Availability

The data presented in the study is deposited in the GEO repository, accession number GSE294616.
