# Supplementary material for: Single-cell RNA sequencing of leukocytes at the maternal-fetal interface in physiological and pathological Nodal-deficient pregnancies
Source: Front Immunol. 2026 Apr 16;17:1611813. doi: 10.3389/fimmu.2026.1611813 (PMC13130886; doi:10.3389/fimmu.2026.1611813)
Supplement: Supplementary file 1 [file DataSheet1.docx]

Supplementary Material

# 1.1 Supplementary Figures


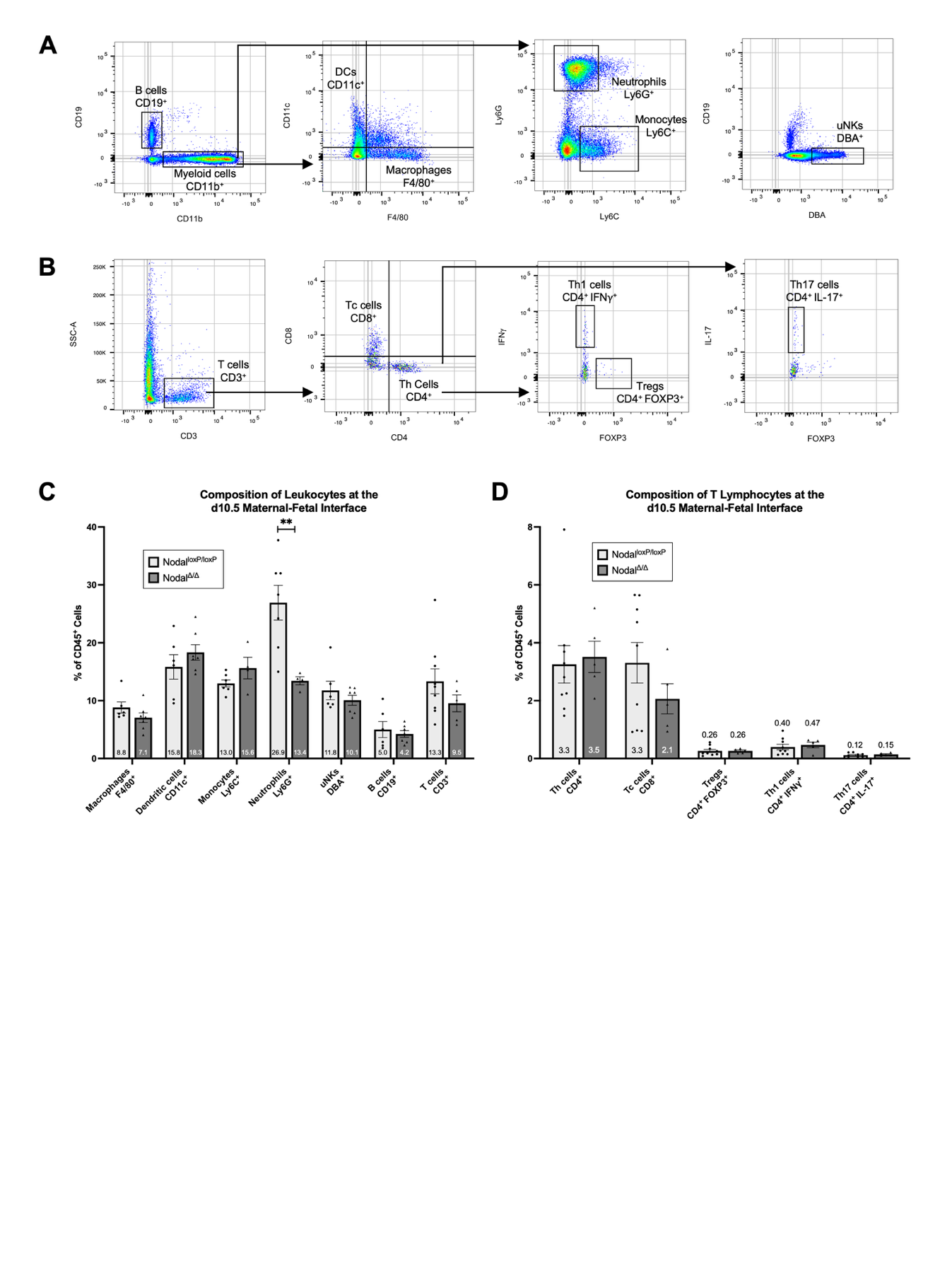


**Supplementary Figure 1: Composition of leukocytes at the d10.5 maternal-fetal interface by flow cytometry**

A) CD11b^+^ myeloid cells at the d10.5 maternal-fetal interface were gated from the live, CD45^+^ population and further gated based on the expression of CD11c (dendritic cells, DCs), F4/80 (macrophages), Ly6C (monocytes) and Ly6G (neutrophils). DBA^+^ uterine natural killer cells (uNKs) and CD19^+^ B cells were gated from the live, CD45^+^ population. B) CD3^+^ T lymphocytes were gated from the live, CD45^+^ population and then further gated based on the expression of CD4 (T helper, Th) or CD8 (cytotoxic T, Tc). CD4^+^ Th cells were gated into additional subpopulations based on the co-expression of FOXP3 (regulatory T, Treg), IFN-$\gamma$ (Th1) or IL-17 (Th17). C) Quantification of leukocytes at the d10.5 maternal-fetal interface showed a significant decrease in the proportion of Ly6G^+^ decidual neutrophils in Nodal^Δ/Δ^ females (Nodal^loxP/loxP^ n=7, Nodal^Δ/Δ^ n=4). There was no difference in the abundance of F4/80^+^ macrophages, CD11c^+^ dendritic cells, DBA^+^ uNKs, CD19^+^ B cells (Nodal^loxP/loxP^ n=6, Nodal^Δ/Δ^ n=7), Ly6C^+^ monocytes (Nodal^loxP/loxP^ n=7, Nodal^Δ/Δ^ n=4) or CD3^+^ T cells (Nodal^loxP/loxP^ n=9, Nodal^Δ/Δ^ n=5). D) Quantification of the T cell subpopulations showed no difference in abundance between groups (Nodal^loxP/loxP^ n=9, Nodal^Δ/Δ^ n=5). Data shows mean ± SEM. **P-value <0.01.


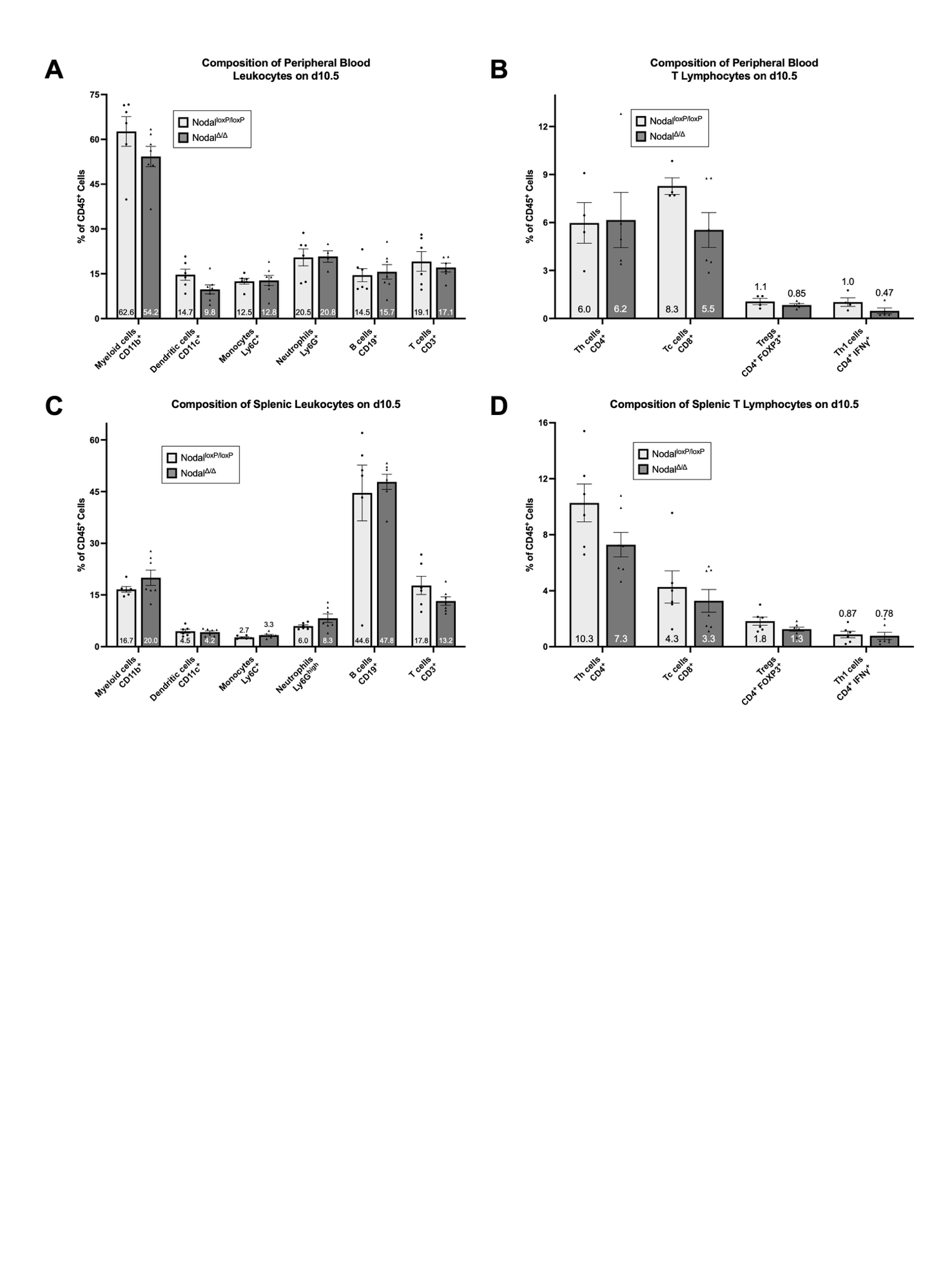


**Supplementary Figure 2: Quantification of matched d10.5 peripheral blood and splenic leukocyte populations**

Matched peripheral blood (A-B) and splenic (C-D) leukocytes from d10.5 pregnant females (Nodal^loxP/loxP^ n=6, Nodal^Δ/Δ^ n=7) were quantified by flow cytometry and showed no significant difference in abundance between groups of any immune cell type. Samples were gated using a similar strategy to cells at the maternal-fetal interface. Data shows mean ± SEM.

**
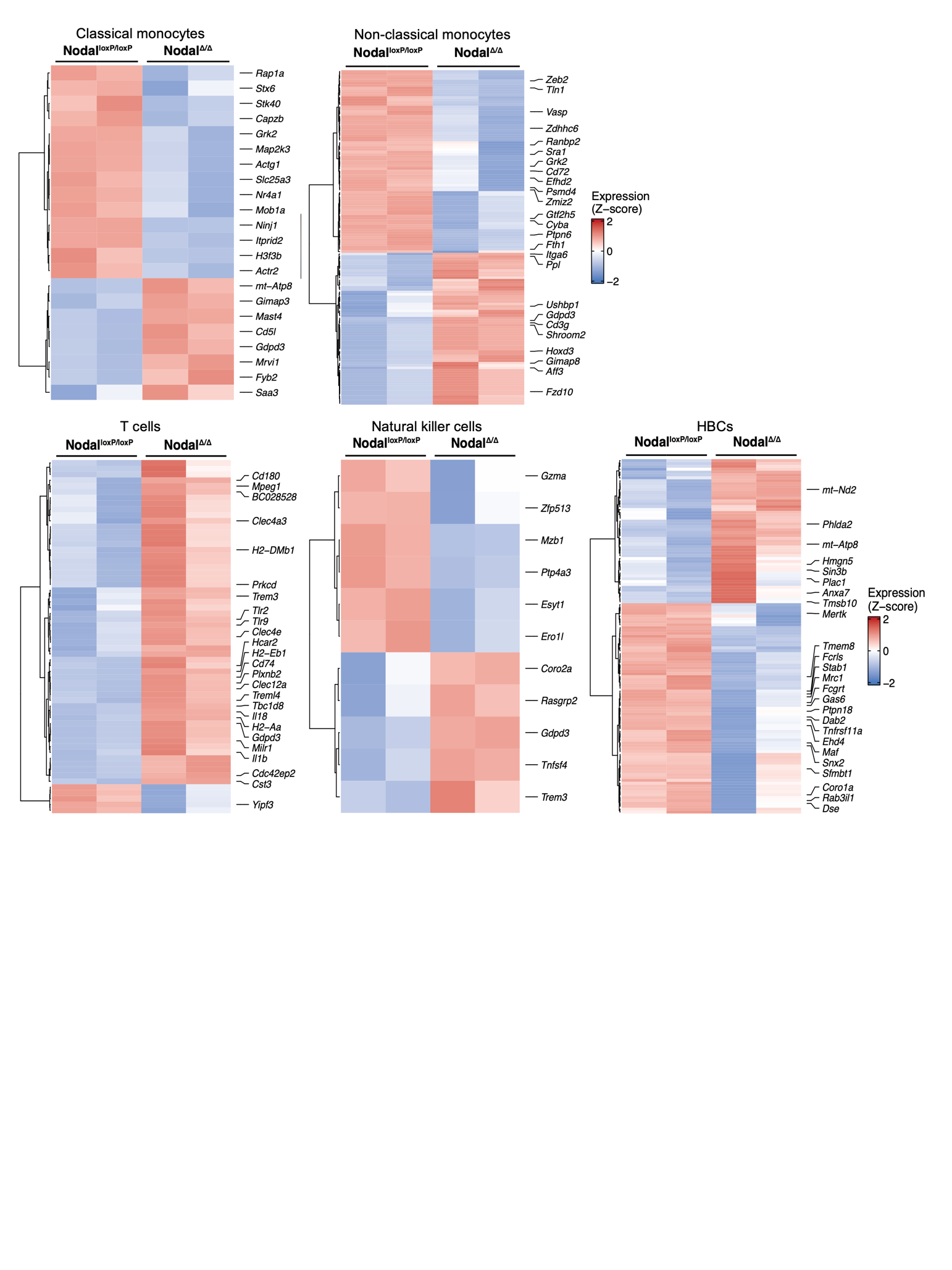
**

**Supplementary Figure 3: Differentially expressed genes in additional immune cell clusters**

Heatmap of significant differentially expressed genes (DEGs) between Nodal^loxP/loxP^ (n=2) and Nodal^Δ/Δ^ (n=2) females in the remaining immune cell clusters at the d10.5 maternal-fetal interface. The top 25 upregulated or downregulated DEGs with an adjusted P-value <0.1 are labelled. There were zero significant DEGs with an adjusted P-value <0.1 in the dendritic cell and B cell clusters.

**
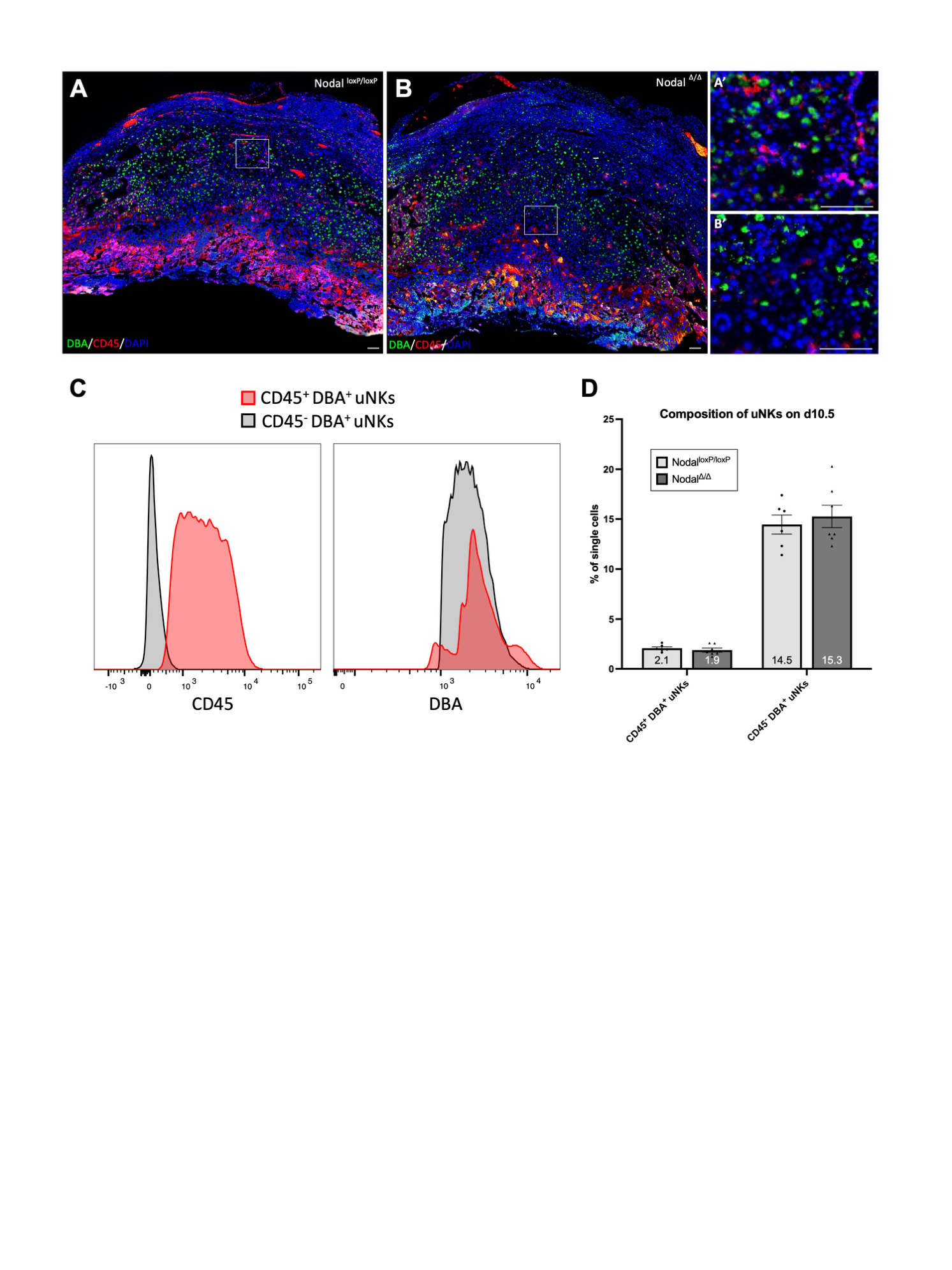
**

**Supplementary Figure 4: Composition of uterine natural killer cells on d10.5**

A-B) Immunofluorescence staining revealed the equal distribution and relative abundance of DBA^+^ (green) uterine natural killer cells (uNKs) in the d10.5 decidua of Nodal^loxP/loxP^ and Nodal^Δ/Δ^ females. Images are representative of 10 implantation sites (Nodal^loxP/loxP^) and 13 implantation sites (Nodal^Δ/Δ^) from three independent pregnancies. A’) DBA^+^ uNKs closer to the mesometrial pole are more CD45^+^ (red). B’) DBA^+^ uNKs closer to the placenta are more CD45^-^. Immunofluorescence images shown at 10X magnification, scale bars indicate 80 $\mu$m. C) Histograms showing the expression of CD45 and DBA in the two populations of uNKs by flow cytometry. D) Quantification of the two uNK populations by flow cytometry revealed that the majority of uNKs at the d10.5 maternal-fetal interface are CD45^-^ DBA^+^, with no difference in overall uNK abundance between groups (Nodal^loxP/loxP^ n=6, Nodal^Δ/Δ^ n=7). Data shows mean ± SEM.

# 1.2 Supplementary Tables

**Supplementary Table 1: List of 34 upregulated genes at the maternal-fetal interface of Nodal^Δ/Δ^ females on d10.5**

| Gene | Fold Change | p-value | Description |
| --- | --- | --- | --- |
| *Il-6* | 6.56 | 0.0179 | inflammatory cytokine |
| *Tlr1* | 6.39 | 0.0097 | pattern recognition receptor |
| *Casp1* | 5.69 | 0.0201 | inflammatory response |
| *Il-1*$\boldsymbol{\alpha}$ | 5.74 | 0.0016 | inflammatory cytokine |
| *Md2* | 4.77 | 0.0117 | TLR4 pathway |
| *Fasl* | 4.55 | 0.0053 | inflammatory response |
| *Ccl12* | 4.15 | 0.0361 | inflammatory chemokine |
| *Cd14* | 3.91 | 0.0051 | TLR4 pathway |
| *Il-2* | 3.72 | 0.0361 | inflammatory cytokine |
| *Apcs* | 3.69 | 0.0376 | inflammatory response |
| *Irf3* | 3.68 | 0.0041 | TLR4 pathway |
| *Mx1* | 3.67 | 0.0003 | inflammatory cytokine |
| *Tlr3* | 3.34 | 0.0016 | pattern recognition receptor |
| *Cd86* | 3.31 | 0.0132 | T cell activation |
| *Il-18* | 3.25 | 0.0357 | inflammatory cytokine |
| *Il-1*$\boldsymbol{\beta}$ | 2.91 | 0.0022 | inflammatory cytokine |
| *Mapk8* | 2.80 | 0.0023 | TLR4 pathway |
| *Crp* | 2.79 | 0.0256 | inflammatory cytokine |
| *Cd40lg* | 2.73 | 0.0467 | macrophage activation |
| *H2-T23* | 2.71 | 0.0072 | antigen presentation |
| *I*$\boldsymbol{\kappa b\alpha}$ | 2.47 | 0.0002 | TLR4 pathway |
| *Jak2* | 2.47 | 0.0104 | TLR4 pathway |
| *Tlr4* | 2.41 | 0.0194 | pattern recognition receptor |
| *Mpo* | 2.27 | 0.0076 | neutrophil response |
| *Mapk1* | 2.24 | 0.0008 | TLR4 pathway |
| *Stat1* | 2.08 | 0.0050 | TLR4 pathway |
| *Cxcl10* | 2.07 | 0.0198 | inflammatory chemokine |
| *Tlr5* | 2.07 | 0.0123 | pattern recognition receptor |
| *Nod2* | 1.95 | 0.0458 | pattern recognition receptor |
| *Cd40* | 1.85 | 0.0173 | macrophage activation |
| *Ddx58* | 1.79 | 0.0458 | pattern recognition receptor |
| *Gata3* | 1.73 | 0.0085 | T cell differentiation |
| *Nf*$\boldsymbol{\kappa b}$*1* | 1.70 | 0.0205 | TLR4 pathway |
| *Irak1* | 1.63 | 0.0213 | TLR4 pathway |

**Supplementary Table 2: Fluorophore-conjugated antibodies used for the identification of immune cell types at the d10.5 maternal-fetal interface**

| Antibody | Fluorophore | Clone | Manufacturer | Cat. No. | Dilution |
| --- | --- | --- | --- | --- | --- |
| Viability dye | e506 |  | Invitrogen | 65-0866-14 | 1:100 |
| CD45 | AF700 | 30-F11 | BioLegend | 103128 | 1:150 |
| CD11b | e450 | M1/70 | Invitrogen | 48-0112-80 | 1:300 |
| Ly6C | APC-Cy7 | AL-21 | BD Biosciences | 560596 | 1:300 |
| Ly6G | APC | 1A8 | Invitrogen | 17-9668-80 | 1:150 |
| F4/80 | PE | BM8 | Invitrogen | 12-4801-80 | 1:150 |
| CD11c | PerCP-Cy5.5 | N418 | Invitrogen | 45-0114-80 | 1:150 |
| DBA | FITC |  | Bio World | 21761015-1 | 1:300 |
| CD19 | BUV737 | 1D3 | BD Biosciences | 612782 | 1:150 |
| iNOS | APC | CXNFT | Invitrogen | 17-5920-80 | 1:150 |
| Arg1 | PE-Cy7 | A1exF5 | Invitrogen | 25-3697-80 | 1:150 |

**Supplementary Table 3: Fluorophore-conjugated antibodies used for the identification of T cell subpopulations at the d10.5 maternal-fetal interface, spleen and blood**

| Antibody | Fluorophore | Clone | Manufacturer | Cat. No. | Dilution |
| --- | --- | --- | --- | --- | --- |
| Viability dye | e506 |  | Invitrogen | 65-0866-14 | 1:100 |
| CD45 | AF700 | 30-F11 | BioLegend | 103128 | 1:150 |
| CD3 | BUV737 | 17A2 | BD Biosciences | 612803 | 1:150 |
| CD4 | APC-Cy7 | RM4-5 | BioLegend | 100525 | 1:150 |
| CD8a | PE-Cy7 | 53-6.7 | Invitrogen | 25-0081-81 | 1:300 |
| TCR$\gamma\delta$ | PerCP-e710 | GL-3 | Invitrogen | 46-5711-80 | 1:150 |
| FOXP3 | FITC | FJK-16s | Invitrogen | 11-5773-80 | 1:100 |
| IL-17 | e450 | 17B7 | Invitrogen | 48-7177-80 | 1:150 |
| IL-4 | PE | 11B11 | BioLegend | 504103 | 1:150 |
| IFN-$\gamma$ | APC | XMG1.2 | BioLegend | 505809 | 1:150 |

**Supplementary Table 4: Fluorophore-conjugated antibodies used for the identification of immune populations in the blood and spleen of d10.5 pregnant females**

| Antibody | Fluorophore | Clone | Manufacturer | Cat. No. | Dilution |
| --- | --- | --- | --- | --- | --- |
| Viability dye | e506 |  | Invitrogen | 65-0866-14 | 1:100 |
| CD45 | AF700 | 30-F11 | BioLegend | 103128 | 1:150 |
| CD11b | e450 | M1/70 | Invitrogen | 48-0112-80 | 1:300 |
| Ly6C | APC-Cy7 | AL-21 | BD Biosciences | 560596 | 1:300 |
| Ly6G | FITC | 1A8 | Invitrogen | 11-9668-80 | 1:150 |
| F4/80 | PE | BM8 | Invitrogen | 12-4801-80 | 1:150 |
| CD11c | PerCP-Cy5.5 | N418 | Invitrogen | 45-0114-80 | 1:150 |
| CD49b | PE-e610 | DX5 | Invitrogen | 61-5971-80 | 1:150 |
| CD19 | BUV737 | 1D3 | BD Biosciences | 612782 | 1:150 |
| iNOS | APC | CXNFT | Invitrogen | 17-5920-80 | 1:150 |
| Arg1 | PE-Cy7 | A1exF5 | Invitrogen | 25-3697-80 | 1:150 |
